# *Ophidiomyces ophidiicola* in Northern Pine Snakes (*Pituophis m. melanoleucus*) in New Jersey: Known-Aged Individuals Indicate Endemic Status, Recovery and Reinfection, and Survival at Least 8 Years Post-Infection

**DOI:** 10.3390/jof12050358

**Published:** 2026-05-13

**Authors:** Joanna Burger, Christian Jeitner, Kelly Ng, Robert T. Zappalorti, John Bunnell, Emile DeVito, David Schneider, David Burkett, Michael Gochfeld

**Affiliations:** 1 Cell Biology and Neuroscience, Rutgers University, 604 Allison Road, Piscataway, NJ 08854, USA; kelng@dls.rutgers.edu; 2 Ecology, Evolution and Natural Resources, Rutgers University, 604 Allison Road, Piscataway, NJ 08854, USA; 3 Center for Environmental Exposures and Disease, Environmental and Occupational Health Sciences Institute, Rutgers University, Piscataway, NJ 08854, USA; mg930@eohsi.rutgers.edu; 4 New Jersey Pinelands Commission, New Lisbon, Pemberton, NJ 08068, USA; christian.jeitner@pinelands.nj.gov (C.J.); john.bunnell@pinelands.nj.gov (J.B.); 5 Herpetological Associates, Inc., Pemberton, NJ 08068, USA; rzappalort@aol.com (R.T.Z.); dschneider@herpetologicalassociates.com (D.S.); burkett1976@yahoo.com (D.B.); 6 Conservation Foundation of New Jersey, Far Hills, NJ 07931, USA; emile@njconservation.org; 7 Rutgers Biomedical and Health Sciences, Rutgers University, Piscataway, NJ 08854, USA

**Keywords:** snake fungal disease, *Ophidiomyces ophidiicola*, disease prevalence, individual differences, sex differences, longitudinal changes in *O. ophidiicola* status, survival post-infection

## Abstract

*Ophidiomyces ophidiicola* (*O. ophidiicola*) causes ophidiomycosis and has deleterious effects in some North American snakes. Studies have suggested that it is endemic in some species, but most studies have been conducted on individuals of unknown age, for only a season or two. This paper fills that gap by exploring prevalence of qPCR-confirmed ophidiomycosis in known-aged Northern pine snakes (*Pituophis melanoleucus melanoleucus*) during a six-year testing period, followed by two years of monitoring survival. Some individuals testing positive for *O. ophidiicola* lived for at least 8 years following initial infection, and their *O. ophidiicola* status could change from positive to negative and back again in successive years, while still seeming healthy. Approximately 85% were positive in at least one year, and overall positivity was 65% in the snakes. Detection frequency was 45% for ventral swabs, and only 23% for head swabs. Of 31 snakes found at least a year after first testing positive, 71% lived at least 3 years, and 23% lived 6 or more years. Females lived longer after testing positive than males, and more females changed from positive to negative than did males. These data help understand infections, recovery and re-infection in individuals, as well as survival of marked individuals, and have implications for endemism and long-term population viability of snake populations exposed to *O. ophidiicola*.

## 1. Introduction

The phrase “Infectious Diseases of Emerging Concern” usually refers to diseases that are novel either to science, a species, or a population, or were naturally occurring yet seem to be suddenly increasing. Thus, “emerging” refers to the concern, not necessarily the disease or disease-causing agent. Infectious Diseases of Emerging Concern have the potential to have adverse lethal and sublethal effects on vertebrates, and in some cases, can devastate populations. Some of the infectious diseases that have been associated with dramatic population declines in vertebrate species include white-nose syndrome in bats (caused by *Pseudogymnoascus destructans*), chytridiomycosis in amphibians (caused by *Batrachochytrium dendrobatidis* and *B. salamaridrivorans*), and ophidiomycosis in snakes caused by *Ophidiomyces ophidiicola* [1,2,3,4]. Several species of bats and amphibians have experienced severe declines worldwide, and there is concern for the potential of local and regional extinctions [5,6,7,8].

Intense interest in ophidiomycosis in snakes exploded in the mid-2010s [9,10,11,12]. *Ophidiomyces ophidiicola* (*O. ophidiicola*) was identified as the causative agent [13,14] and is increasingly identified in many parts of North America (e.g., Texas [15], Canada [16]), as well as in Europe and Asia [17]. Clinical signs of ophidiomycosis include dark and ragged lesions (sores), facial swellings, dermal ulcerations, pustules, blistering, white residue, skin abnormalities and skin ulcerations [2,18,19]. However, clinical signs (e.g., skin lesions) that are not tested by qPCR (Quantitative Polymerase Chain Reaction) may, or may not, be caused by *O. ophidiicola*. Identification of ophidiomycosis is usually done by swabbing ventral surfaces, heads, and lesions, and presence of the fungus is determined by qPCR tests for *O. ophidiicola*.

The demise of some viper populations due to *O. ophidiicola* has led to overall concern for the effect of ophidiomycosis on snakes [2,20]. Clark et al. [20] reported a loss of nearly half of the timber rattlesnake (*Crotalus horridus*) population that was associated with ophidiomycosis. However, the case of ophidiomycosis is quite different from other virulent, fungal diseases because, although its presence has been identified in more places, with in species of snakes, the adverse effects of ophidiomycosis on most species and populations of snakes have not increased [18,21,22,23]. That is, although *O. ophidiicola* has been associated with population declines in some species [20], for most species that have tested positive, significant adverse effects on population levels have not been identified, despite numerous reports of clinical signs of ophidiomycosis [10,23,24,25,26,27]. In some species there are, however, non-lethal changes in hormone levels, and abnormalities in reproductive physiology, basking, and thermoregulation [2,9,11,14,19,27,28,29]. The large number of studies of snakes showing clinical signs on their skin have not been linked to mass mortality or population declines [16]. This has led us and others to propose that ophidiomycosis is endemic in many populations of snakes [18,22,23,25,26]. In addition to considering whether the presence of ophidiomycosis in snake populations is endemic, the question of seasonal and yearly variations in prevalence in populations is important for understanding ophidiomycosis and its possible effects. Some studies have shown seasonal differences in prevalence of ophidiomycosis, with prevalence decreasing from egress from hibernation to the fall [19,20]. Lind et al. [28] reported that prevalence is high in winter and low in summer in pygmy rattlesnakes (*Sistrurus miliarus*); they used clinical symptoms (and not qPCR) and collected data only for two years.

Some difficulties of some previous observations are that individuals are usually not recaptured for many years, the age and sex of individuals is unknown, and survival, recovery or death post-infection are unknown [10]. Many studies have stated that they did not find moribund individuals, for example, [17]. There are dozens of papers on prevalence of *O. ophidiicola* in snakes, but none that follow known-aged individuals over many years. Many papers do not identify snake ages, while others identify the snakes as juveniles or adults [15,30,31,32] or by size [4,16]. Most studies test snakes for *O. ophidiicola* without regard for individual identity, and data are presented for the populations tested. This is partly a result of the difficulty of finding snakes in general, finding snakes as hatchlings when they can be accurately aged, and having long-term studies where the same snakes are followed for years [23,25,33].

This lack of individually marked snakes followed for more than a few years results in not knowing whether ophidiomycosis is progressive or whether individual status changes from year to year in individuals. Once positive, how often does a snake ever shift to negative (and later back to positive), and are there sex differences in their transition status? Likewise, does severity change in individuals, as indicated by more severe lesions in succeeding years, and what is the rate of survival following an initial infection (testing positive by qPCR)? Some studies report that some snakes test both positive and negative within a season but there are few data [15,28,34]. We suggest these aspects are essential to understanding the role of ophidiomycosis in survival of individual snakes and the potential role of ophidiomycosis in population dynamics. The lack of long-term *O. ophidiicola* studies with marked individuals is a serious conservation concern [15].

In this paper, we examine the prevalence of ophidiomycosis in individual Northern pine snakes (*Pituophis melanoleucus melanoleucus*) in the New Jersey Pine Barrens. Northern pine snakes are listed as threatened in New Jersey, and are endangered, threatened, or declining in all other states where they occur [35]. The New Jersey population is separated from other Northern pine snakes living in the southern United States by hundreds of kilometers [36]. Our objectives were to examine ophidiomycosis status of known-age, known-sex individuals sampled late in hibernation, sometimes called brumation, to determine detection frequency of *O. ophidiicola* on different areas of the body, in succeeding years, as a function of sex and age over the period from 2018 to 2023. Snakes were then monitored in 2024 and 2025 for survival. We examined the frequency of changes in *O. ophidiicola* status in pine snakes switching from positive to negative, and negative to positive, as well as sex and age effects. We predicted that once positive, snakes would remain positive the next year, that there would be no differences in the prevalence of *O. ophidiicola* in females and males, and that *O. ophidiicola* frequency would increase with age. We also considered that with successive years of being positive, snakes might have more sores; that is, the number of sores may be an indication of increasing severity. The advantages of testing snakes while in hibernation is that we can reliably find individuals, they are all tested at the same stage and time period, and none of them have eaten since they entered hibernation in the fall. In these hibernacula, there is a high correlation between the presence of *O. ophidiicola* on the snakes and in the soil in the chamber where they hibernated [37]. Prevalence in New Jersey Northern pine snakes that were tested during late hibernation ranged from 46% to 100% a year over a 6-year period [23,25]. The value of the present study is that it provides data on prevalence of ophidiomycosis in known-aged pine snakes, assessing *O. ophidiicola* positivity over a 6-year period, followed by monitoring survival over an additional two years. The presence of ophidiomycosis was examined at the same time for all snakes in hibernacula (late winter). These data are not available for any other snake species in the world.

## 2. Materials and Methods

### 2.1. Study Species

In New Jersey, Northern pine snakes dig their own nests and largely construct their hibernacula, where there can be 2 to over 30 snakes [38,39]. Pine snakes dig their own hibernation chambers from a central chamber; some chambers have more than one pine snake coiled together. They enter hibernacula in October and November, where they remain until late March or early April [40]. In late August or early September, hatchlings emerge from nests and seek hibernation sites. Philopatry to hibernation sites is high in pine snakes; females display higher fidelity than do males [41]. Poaching, road kills, and off-road vehicles are major causes of population declines [42]. To avoid poaching, exact locations of hibernacula are not disclosed. As a threatened species, they are not common, and sample sizes are lower than can be found in studies of common or abundant snakes. However, the advantage of being able to find hibernacula, excavate them, and find individuals in succeeding years is that it provides the opportunity to examine prevalence of ophidiomycosis over several years, and addresses individual differences in *O. ophidiicola* We have conducted hibernation, growth, and survival studies on pine snake hibernacula for over 40 years by digging them up [23]. We designate a pine snake that hatched in August or September as age 0, and when encountered during excavation of their hibernaculum in early spring, they are designated a one-year-old. The following March, when the snake is about 18 months old, we designate it as a 2-year-old. The ages of all hatchlings and second-year snakes can be identified; there is no overlap in sizes of first- and second-year snakes [38].

In 2018 we began our ophidiomycosis study, whereby we swabbed each snake’s head extensively and did a swab of the entire ventrum. In 2019 we expanded to swabbing individual sites (e.g., head, ventrum, cloaca, all lesions) to determine frequency of *O. ophidiicola* in different areas on the snake’s body [25]. For this paper we examine individual pine snakes that were tested at least twice for *O. ophidiicola* from 2018 to 2023; subsequently they were monitored for survival for an additional two years (2024, 2025) while in hibernation. All snakes reported in this study (N = 41) were captured and tested more than once; 54 others were captured but not relocated.

### 2.2. The Sampling Protocol

Our overall protocol was to locate snakes in hibernacula in late February or early March and move them to a sampling station for swabbing. As with any study in the wild, we did not locate all snakes every year, and in the present study some snakes were first found after the first year (2018) of *O. ophidiicola* testing and became part of the study in later years. While this represents a limitation of the current study, few ophidiomycosis studies have followed individuals from year to year.

Snakes were swabbed, then checked for PIT tag identification. All swabs were labeled by snake and type of sample and were placed in an ice chest for later freezing, and analyzed by qPCR. All personnel removing snakes from the soil, transporting snakes to the *O. ophidiicola* sampling station, and taking swabs wore disposable nitrile gloves; all personnel changed gloves between snakes. Each snake was given a field number when it was removed from its hibernation chamber. All snakes were returned to their hibernaculum after sampling on the same day. All handling of snakes and sampling procedures were approved by the Rutgers University Institutional Animal Care and Use Committee (Protocol 86-017, renewed every three years) and appropriate state permits.

**Figure 1:**
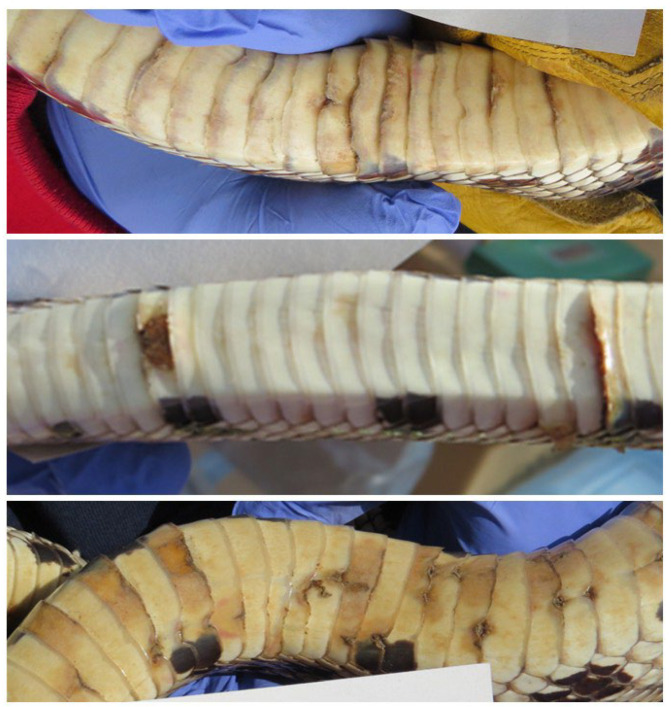
Examples of lesions on pine snakes that tested positive for *O. ophidiicola* (qPCR).

At the *O. ophidiicola* sampling station, swabs were taken from the ventrum (whole body), head, cloaca, and each abnormal area or lesion (sometimes called hibernation sores). Ventral swabs were taken by firmly running a polyester-tipped swab downward from the neck to anterior of the cloaca using a single continuous pass. We swabbed the head because it has been found to be the source of severe infections in some snake species [43]. Lesions included any discolored or elevated scales and abraded scales with discolored ragged margins (Figure 1). Swabs were premoistened with 20 µL of sterile deionized water. Swabs were placed in sterile tubes, and all swabs from each snake were put into a bag marked with the snake number, placed on ice in the field, and stored at −30 °C at Rutgers University. After samples were collected for qPCR analysis, snakes were transferred to a separate station to be measured, weighed, and given a PIT tag if they were previously unmarked (AVID Identification Systems, Inc. Norco, CA, USA). Between one hibernation complex and another, shovels and other equipment were washed with bleach. Further methods are described in Burger et al. [23,25]. Thereafter, snake samples were transferred to Rutgers University for later analysis.

### 2.3. Determining Ophidiomyces ophidiicola via qPCR (Quantitative Polymerase Chain Reaction)

Nucleic acid was extracted from the swab samples and screened for the presence of *O. ophidiicola* using a specific qPCR targeting the internal transcribed spacer region of the fungus as described by Bohusky et al. [44]. We defined a sample as positive for *O. ophidiicola* if it had 15 or more copies of the target DNA, as determined based on standard curves included on each qPCR run. If any swab on a snake was positive, we considered the snake to be positive. All analyses for detection of *O. ophidiicola* were conducted under the direction of J. M. Lorch (U.S. Geological Survey—National Wildlife Health Center in Madison, WI, USA) and were conducted in his laboratory. We did not examine internal tissues for disease, as no pine snakes were found dead during this study.

### 2.4. Statistical Analysis

We used the following non-parametric tests: a Mann–Whitney U Test for continuous variables, and Fisher’s Exact Test (SAS 2020, PROC NPAR1WAY) for determining differences in discrete variables (e.g., female vs. male) [45,46]. These non-parametric tests were used because they are more conservative and are best suited for small datasets with binary outcomes [47]. A Chi-square Test of Homogeneity was used to evaluate the differences between males and female survival. A value of *p* < 0.05 was considered significant, although with small sample sizes, any *p* value below 0.10 would likely be more significant than with larger sample sizes. Snake-years equals the sum of the number of snakes X the number of years in which each snake was tested. If snake 1 was tested for 6 years, and snake 2 was tested for 3 years, snake-years would equal 9.

## 3. Results

### 3.1. General Variation in the Dataset

In this study we report on data from 22 female and 19 male known-aged snakes that were qPCR tested more than once. An additional 54 snakes were tested but were not found again in hibernation; they were mainly 1–2-year-olds that had low survival. There was variation in individual patterns of *O. ophidiicola* among the 41 snakes considered in this paper. Table 1 presents sequential data for a few individual snakes that illustrate a variety of patterns for some of the longest-lived snakes. Each of the examples has an explanation of *O. ophidiicola* testing, status, and subsequent survival of the snakes. In Table 1 we also note the years during which a snake was missing with an M. Where a cloacal sample is blank it means that the qPCR sample was on the line between being positive and negative. These examples represent some of the snakes with the longest testing records to illustrate the variation between years for the same individual. Both males and females are shown in order to illustrate that males and females in the study can live for many years post-infection.

Snakes differed in years they were positive, in tissues that were positive, in whether they had lesions (and if they were positive), and in whether they were missing from the hibernacula in some of the intervening years. Some snakes were negative for the first year or two of testing, some were positive and remained positive every year of the study, and some switched back and forth.

The explanations for each snake’s pattern are presented for each snake under the notes in Table 1. In addition to the information given, the following should be noted: (1) some snakes that were classified as positive had only one lesion test positive, and other tests were negative; (2) snakes were found in the hibernacula in the early years of the ophidiomycosis study could disappear and not return for up to 5 years; and (3) head and cloaca samples had a lower rate of positivity than lesions.

### 3.2. Behavior of Known-Aged Pine Snakes Found More than Once During Ophidiomycosis Study

In the previous section, the histories of some individual pine snakes were described to illustrate the dataset. Examining the 41 snakes that were tested two or more times provides a picture of the relationships between testing positive for *O. ophidiicola* and age, sex, and number of lesions. We summarize the data on individual snakes for the main characteristics in Table 2. Both the overall means, and the means by sex are provided to allow comparisons with most studies that do not identify the sex of the snakes examined for ophidiomycosis. The endpoints examined are grouped and numbered for descriptive purposes. Section 1 lists the number of snakes examined. Snakes could be tested up to six times, and this number decreased throughout the study for individuals first tested later in the study (2019–2023). That is, a snake first tested for ophidiomycosis in 2020 could not have as many tests as one tested at the beginning of the study (2018).

Section 2 (Table 2) lists the mean number of years snakes were tested, and the total number of test years (sum of the number of years each snake was tested), for pine snakes. Since snakes were captured and tested while in hibernation and all were tested, the relationship between males and females partly reflects the philopatry of snakes. Most snakes (35 of 41 snakes = 85%) tested positive during at least one year in their test series (Section 3). However, the percentage of snakes testing positive overall (the measure usually reported for snake populations where snakes are unmarked) drops to 65% (Section 3). That is, the overall prevalence rate for Northern pine snakes that is comparable to other studies in the literature is 65% over our 6-year study, because the 41 snakes were tested an average of 2.8 times (41 × 2.8 = 115 snake-years). The overall rate included any swabs that tested positive.

Ventral swabs had a lower positivity rate (45% overall), and head swabs were lower still (21%, Section 4). Section 5 shows the percentage of snakes that had no lesions/year (after the first year they tested positive for *O. ophidiicola* in qPCR). That is, some snakes tested positive without having any lesions. It also shows the mean number of lesions per snake, and that overall, 87% of all lesions were positive (Table 2, Section 5).

Section 6 (Table 2) provides data on a measure not previously computed in the ophidiomycosis literature for known-aged and sexed snakes—the *O. ophidiicola* status from one year to the next. We had predicted that once positive, a snake continues to be positive. However, that was not the case for pine snakes in this study. Table 2 shows the percentage of times snakes shifted from one status to another. The sexual differences were clear in this measure; females were more likely than males to go from positive to negative (Table 2, Section 6). Only one male’s status switched from positive to negative during the period of *O. ophidiicola* testing. Further, males averaged 1.2 status changes, while females averaged 2.3 (*p* > 0.04).

**Table 2:** Summary of testing, prevalence of *O. ophidiicola* and temporal status of qPCR testing for snakes tested 2 or more times (e.g., testing positive for *O. ophidiicola*). Mann–Whitney U Test was used on continuous variables because of the small sample size and non-normal distribution; Fisher’s Exact Tests were used on discrete variables (male, female). NS = not significant; Section # = section number.

Section #	Characteristics	All	Male	Female	*p* Values
1	Snakes tested 2 or more times	41	19	22	
2	Mean number of years each snake was tested for SFD	2.8 ± 0.2	2.5 ± 0.2	3.1 ± 0.3	NS
	Total number of years tested (combined for the 41 snakes)	115	45	70	
3	Percentage of snakes that tested positive at least once	85%	79%	90%	NS
	Overall % of snakes that tested positive for *O. ophidiicola* over the 6 year period	65%	62%	67%	NS
4	Percentage ventral swabs that tested positive	45%	55%	38%	<0.08
	Percentage of head swabs that tested positive	23%	20%	25%	NS
5	Number of snake years ^a^ in which snakes had no lesions	27	7	20	NS
	Percentage of snake-years ^a^ in which snakes had no lesions	23%	16%	28%	NS
	Mean number of lesions/snake	3.2 ± 0.27	3.4 ± 0.5	3 ± 0.34	NS
	Percentage pf positive lesions/all lesions	87%	84%	89%	NS
6	Number of status categories from one year to the next ^b^	73	22	51	
	Negative to negative	16%	17%	16%	0.04
	Negative to positive	23%	27%	21%	
	Positive to positive	46%	55%	43%	
	Positive to negative	14%	<1%	20%	

^a^ Snake-years = the number of snakes examined X the mean number of years snakes were tested. ^b^ For example, a snake with 5 years of *O. ophidiicola* qPCR testing has 4 status changes. A snake with only 2 years of data has 1 status change. A higher number of status changes reflects that females had longer chains of years of qPCR testing than did males.

Additional conclusions drawn from the dataset include the following. (1) Some snakes sampled for *O. ophidiicola* (ophidiomycosis) only twice were negative both times, while others were positive both times. (2) Every snake sampled more than twice had a least one lesion. (3) Most snakes sampled more than twice were positive at least once. (4) Five females and one male always tested negative (and are thus not on the graphs where snakes tested positive); three females and one male that were tested twice (and might otherwise be part of this study) tested positive after testing negative and were not tested again. Thus, for the analysis where we examine snakes after testing positive, the sample size is 31 (41 minus the 10 snakes just described). Further descriptions of sex, age, and status changes are given below.

### 3.3. Prevalence of Ophidiomyces ophidiicola, Age First Tested, and Years Tested

**Figure 2:**
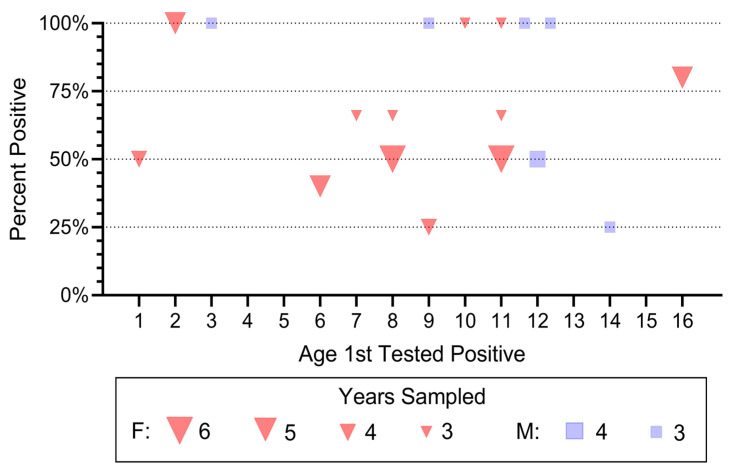
Effect of age at first testing on percentage of times individual pine snakes tested positive for *O. ophidiicola*. This includes only individuals tested 3 or more times (N = 12 females and 6 males). There were no significant age or sex differences, although females tended to be sampled over a greater number of years (*p* < 0.08).

We examined the percentage of snakes that tested positive for *O. ophidiicola* and had at least two years of data following a positive test for *O. ophidiicola* (Figure 2). We make two observations from this sample: (1) more females were in the hibernacula and tested positive than males, and (2) after testing positive, snakes often tested negative far less than 100% of the time. This illustrates the data reported in Table 2.

### 3.4. Lesions

Lesions are one of the clear clinical signs of ophidiomycosis and the one often used in other studies to determine which snakes to test. When a pine snake tested positive, they usually had some lesions (Table 2). Overall, in only 23% of snake-years in which snakes tested positive did they have no lesions. Further, there were no sex differences in the number of lesions, or the percentage of lesions that were positive (87% overall) (Table 2).

#### 3.4.1. Abundance of Positive Lesions in Successive Years

**Figure 3:**
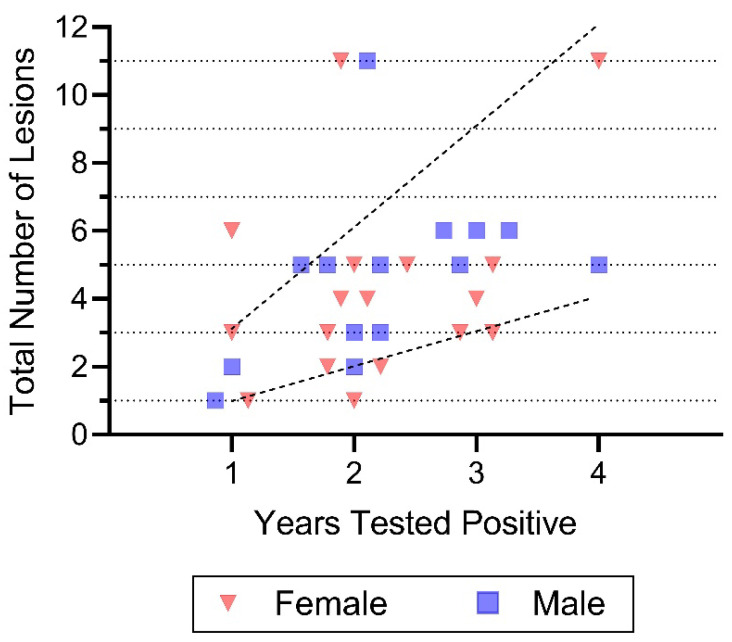
Total number of lesions on pine snakes as a function of the number of years snakes tested positive for *O. ophidiicola*. Each symbol is one snake. The bottom dotted line on the graph equals the number of lesions a snake should have if it had 1 lesion the first year and tested positive 4 times (it should have 4 lesions if it was equally infected each year). The upper dotted line indicates that if a snake had 3 lesions the first year in which it tested positive, it should have 12 lesions overall if it were tested for four years. Most snakes did not have more lesions in successive years than when they were first tested. There was no sex difference in this endpoint, but there is individual variation among snakes.

We examined the question of whether snakes that tested positive in successive years had more or fewer lesions (Figure 3). This was undertaken because looking at the data presented in Table 2, the highest positive rate for *O. ophidiicola* was for lesions and the number of lesions varied by snake. Having more lesions/year in succeeding years might indicate that ophidiomycosis is getting more severe. If the disease is remaining similar in severity for snakes testing positive in successive years, there might be about the same number of lesions/year. That is, if a snake has one lesion the first year it tests positive, and is tested for 3 additional years, the total number of lesions (to keep the number the same), should be four after testing for 4 years (1 each year). Similarly, a snake tested for 4 years with 3 lesions in the first year should have a total of 12 lesions to have the same level of infection. However, this was rarely the case (Figure 3). In successive years, the number of lesions/snake seemed to decrease (Figure 3). That is, the average number of lesions/snake/year was 2.6 when they tested positive only once, it was 2.2 when they tested positive twice, and averaged 2 when they tested positive both three and four times. Thus, there is a clear tendency for the number of lesions to decline with successive years for pine snakes that tested positive for *O. ophidiicola*.

#### 3.4.2. Effect of Snake Age on Number of Lesions

**Figure 4:**
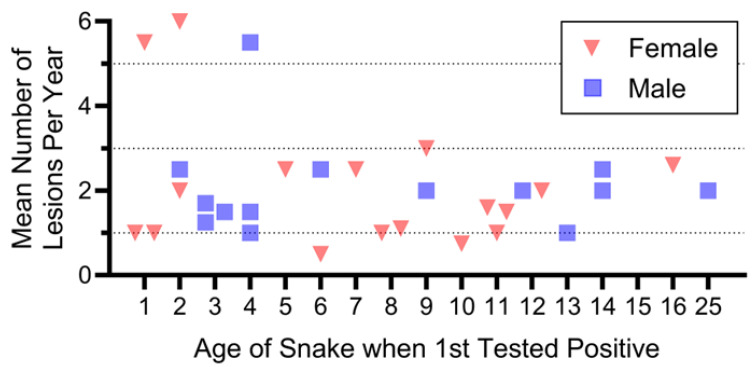
Mean number of lesions/snake/year as a function of the age when they were first tested for *O. ophidiicola*. Except for three snakes, all averaged 1–3 lesions/year, and there were no sex differences.

We expected that older snakes testing positive might average more lesions than younger snakes, either because old snakes are more susceptible, or because they have had longer to acquire lesions. However, for all but three snakes, the mean number of positive lesions/year was usually less than three (Figure 4). Three individuals with five or six lesions/year were 1, 2, and 4 years old when first tested. There were no sex or age-related differences in the mean number of lesions/snake/year as a function of the age when they first were tested. Thus, remarkably, older snakes did not have more lesions.

### 3.5. Status from Year to Year

One key question is whether snakes continue to test positive for *O. ophidiicola* after first testing positive (e.g., for successive years). We computed the status from one year to the next for all pine snakes tested for *O. ophidiicola* at least twice while in hibernacula. Thus, for each snake, the number of possible shifts was one less than the number of times a snake was tested after it first tested positive. The *O. ophidiicola* status could remain the same or change in either direction. We propose that the direction of the change should be independent of the number of samples per snake, since at every point, the status the next year could be any of the options.

**Figure 5:**
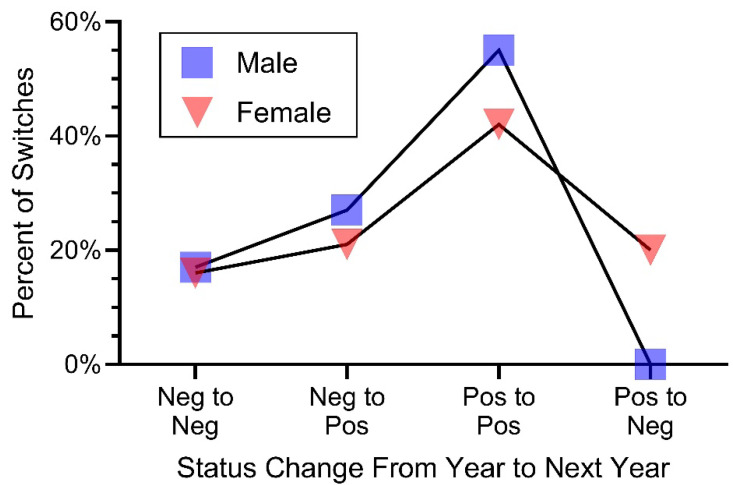
Percentage of status changes in *O. ophidiicola* in pine snakes from one year to the next. Neg = negative, and Pos = positive according to qPCR tests. Significantly more females switched from positive to negative than did males.

The possible transitions in status were negative to negative, negative to positive, positive to positive, and positive to negative. The key question is as follows: Do snakes that are positive remain positive? Table 1 shows the percentage of changes from one year to the next. These shifts are illustrated in Figure 5: significantly more females changed from positive to negative than did males (*p* < 0.04, Table 2). During our study, only one male shifted from positive to negative.

### 3.6. Survival Following Ophidiomycosis

**Figure 6:**
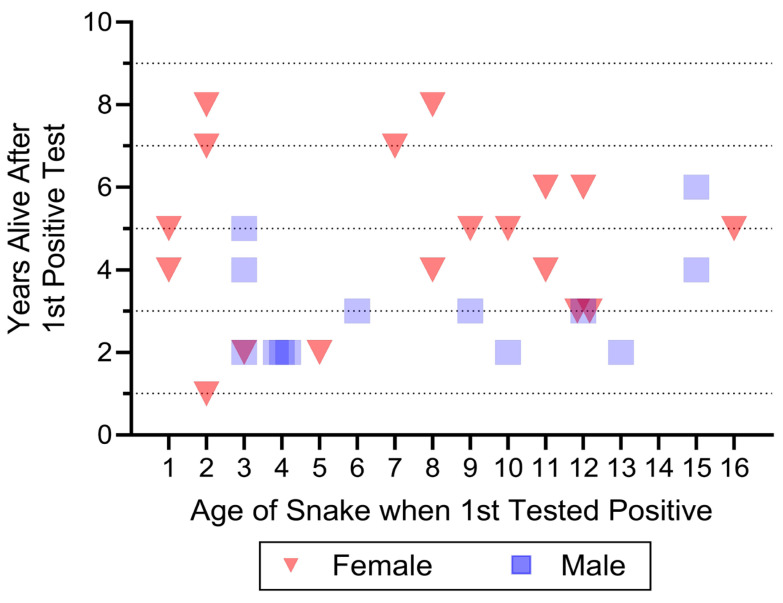
Years male and female pine snakes were still alive after first testing positive for *O. ophidiicola* (N = 31). This is a minimum since snakes could still have been alive when our study ended (2025). Each symbol represents one snake; there is some overlap in snakes on the graph. There was a significant sex difference, with females living longer than males after testing positive (*p* < 0.02).

The length of time (e.g., years) that snakes were alive following their first positive test is shown in Figure 6. This does not include: snakes that only tested negative; snakes that only tested positive after testing negative but were never seen again; and snakes that were encountered only once (whether negative or positive). The variation in initial age of the snakes reflects their participation in a long-term study of known aged snakes. Some snakes that were recaptured for only 1 or 2 years were found as hatchlings (at any time from 2018 to 2021), and others were located later in the 6-year ophidiomycosis study.

Upon inspection, Figure 6 indicates that: (1) some snakes were alive 8 years after first testing positive, (2) snakes first tested positive for ophidiomycosis at all different ages (0–16 years), and (3) females were found alive for significantly longer following first testing positive than were males (*p* > 0.02) (Figure 6).

First, and foremost, these are the first data that show that some snakes can live at least 8 years after first testing positive for *O. ophidiicola*. Second, Figure 6 shows that snakes testing positive at 2 years old can live at least 8 years after testing, snakes tested at 7 or 8 years can live at least 7 or 8 more years, and even snakes first tested at 15 or 16 years old can live at least 4–5 years after first testing positive. Two snakes not on the graph deserve mention: (1) one male tested negative at both 8 and 13 years of age (and was not found in between), and (2) one male tested negative at 24 years old, and tested positive at 25 years, but was not found thereafter (this is a longevity record for any wild *Pituophis* snake, according to Burger et al. [35]). Any of these snakes shown on Figure 6 may still be alive in 2026 or later, particularly those that were tested when they were under 5 years of age.

## 4. Discussion

Understanding the prevalence and severity of ophidiomycosis in a range of snakes is critical for protection and conservation of snakes in general, as well as for threatened or endangered species such as pine snakes. Recently, ophidiomycosis was reported in the Louisiana pine snake (*Pituophis ruthveni*) [48]. The Louisiana pine snake is a federally threatened species according to the USFWS [49]. Understanding the prevalence of ophidiomycosis within a spatial context is necessary to determine if the fungus is endemic or is still in the spreading phase, which identifies its potential importance not only for future population dynamics, but also for developing responses or strategies to conserve vulnerable populations. Studies of wild snakes can complement laboratory studies where snakes are deliberately infected with *O. ophidiicola*. We suggest that it is possible, however, that effects in the laboratory may be more severe than for snakes in the wild that have natural and familiar environments, and that laboratory snakes may have more behavioral adaptations such as changing thermoregulatory behavior, temporary egression from hibernation, and even more frequent shedding because of physical structures that relieve disease symptoms. For example, in the wild there are more numerous and diverse places to aid snakes in shedding their skin, which is known to often result in clearing of clinical signs; they can move over greater distances, thus not infecting damp soil where they may rest periodically, which could reduce their clinical signs. Understanding prevalence, infection severity, and recovery and reinfection provides context for recurrence of clinical signs and positive qPCR testing. Documenting factors associated with *O. ophidiicola* positivity and long-term survival in wild snakes will contribute to our overall understanding of *O. ophidiicola*’s effects on wild populations and potential conservation concerns.

The overall objective of the present research was to examine the long-term survival of individual, known-aged pine snakes after they first tested positive for *O. ophidiicola*, as well as identifying correlations with age, sex, and years of ophidiomycosis prevalence as determined by qPCR testing. These results come with the caveat that many of the results depend on age of first testing, and that snakes were first tested at different years in our testing period (e.g., 2018–2023). Additionally, Northern pine snakes in New Jersey Pinelands are threatened (rare with low populations) [36,50]. As they are both a rare and secretive snake, our sample sizes are low. The advantage of this study is that all snakes were aged, sexed, and monitored for many years [35,39]. Thus, once our *O. ophidiicola* study was initiated in 2018, we had known-aged individuals ranging from hatchlings up to 24 years. Each snake is valuable; this is the only study with data on known-aged individuals that were tested for *O. ophidiicola* over a 6-year period (and monitored for an additional 2 years). We suggest that only by examining individuals over time can we ascertain whether there are differences as a function of year, age, and sex, whether the fungus is endemic in the population, and whether there are long-term effects on individuals and populations.

The main conclusions of our Northern pine snake study are: (1) Known-aged snakes that lived at least one year beyond first testing positive had the same positivity rate (65%) as the overall population of snakes tested in our hibernacula. (2) There were few sex differences in rate of positivity for *O. ophidiicola*. (3) There were differences in positivity as a function of sampling site; head swabs had the lowest percentage positivity (23%) and lesions had the highest (87%). (4) Significantly more females switched from positive to negative. (5) Females were encountered for more years after testing positive for *O. ophidiicola* than males in this present study (even though in this population, there was no overall sex difference in longevity over the 40 years of our work [35]). (6) Some pine snakes can live at least 8 years after first testing positive for *O. ophidiicola* (some of these snakes are likely still alive). In our long-term, 40-year study, males show less fidelity to specific hibernacula than females, but over time the survival is similar [35]. Thus, we did not expect differences in survival in the snakes examined for *O. ophidiicola* in the last 8 years of the study when we tested for *O. ophidiicola*. However, males are less faithful to specific hibernacula as they age [35]. These data provide the first evidence of long-term survival in individual snakes following testing positive for *O. ophidiicola* that survival post-infection is equally high at different ages, and that females significantly more often shifted from positive to negative than males, perhaps indicating higher resistance. Below we discuss *O. ophidiicola* prevalence, factors associated with *O. ophidiicola* positivity, endemism of ophidiomycosis in pine snakes, and survival and longevity.

### 4.1. Prevalence and Transmission

We found that very few swabs on the head tested positive. This is an important finding because some authors have suggested that infections centered on the head lead to internal infections and mortality, particularly in laboratory studies where snakes were inoculated [2]. In the wild, Clark et al. [20] found that many rattlesnakes had skin lesions around the head and chin, and one that died had severe fungal infections in the mouth. *Ophidiomyces ophidiicola* on the head and mouth may lead to more severe infections (than skin lesions, for example) and mortality, suggesting that more field studies should examine morphological locations of *O. ophidiicola* to understand vulnerability to the fungus.

Two other issues that should be considered with transmission and prevalence of ophidiomycosis are their microbiome and cryptic connections. The skin microbiome should be examined in Northern pine snakes because differences might account for susceptibility and vulnerability. For example, Hill et al. [51] noted that 58 bacterial and fungal strains were isolated from the skin of timber rattlesnakes and Northern black racers (*Coluber constrictor*), and the microbiome did not differ between the two species. Hill et al. [51] suggested that the microbiome might contribute to differences in susceptibility, and we suggest that this should be examined in species such as the Northern pine snake that have high prevalence of the fungal disease in individuals over time, without apparent permanent effects ([25,35], this study).

We assume that *O. ophidiicola* in northern pines is spread from pine snake to pine snake while they are in hibernacula, and from the soil to pine snakes; we demonstrated this with snakes and soil tested in our hibernacula [25,37]. However, other species co-habit our pine snake hibernacula systems, including black racers, corn snakes (*Pantherophis guttata)* and timber rattlesnakes. Sometimes pine snakes are in the same chamber as these other species, where transmission could occur. Transmission could go in either direction. Rattlesnakes, the species known to have high prevalence of *O. ophidiicola*, can be severely affected and die [20], which did not occur in our pine snake hibernacula until 2010. A female rattlesnake gestated on the surface of one of our major dens in the late 2010s and early 2020s, and newborn rattlesnakes during the same time spent several months going in and out of two of our hibernacula. There might have been some transmission from rattlesnakes to pine snakes. These intraspecific transmissions would be through cryptic connections not previously considered [4]. Pare and Sigler [52] mentioned in passing that there could be “spill over” in infections from one snake species to another in populations where different species come together. Some racers, corn snakes, and rattlesnakes tested positive when we found them in our hibernacula (Burger, Unpublished data [35]). We suggest that *O. ophidiicola* was not very common based on a general lack of lesions on pine snakes before that date (Burger, Unpublished data [35]).

### 4.2. Factors Affecting Prevalence: Age Class, Sex, and Year

Several additional factors might explain the prevalence of ophidiomycosis in snakes in general, and pine snakes in particular, including sex, age, and size, as well as year of sampling. The latter could account for local outbreaks [53], or the apparent appearance of outbreaks. For example, in one year of our study, 100% of pine snakes tested positive, but this was not the case in other years [23].

Some studies report no difference in prevalence of ophidiomycosis as a function of size, snout–vent length, or weight [54], but most reports in the literature were conducted in only one or two years, which does not allow for analysis of yearly differences. Snout–vent length is perhaps the easiest to examine because any snake tested could be measured, and in most cases, size was used to categorize snakes as adults or juveniles [16,28,53,55]. Dillion et al. [16] found that prevalence and lesions increased in longer Eastern foxsnakes (*Pantherophis vulpinus*), and they suggested that older snakes had more opportunities to encounter the fungus. These snakes, however, were neither sexed nor aged. When researchers found a difference in ophidiomycosis, most studies report that adults had higher prevalence rates than juveniles [15,53]. This was not the case in our studies.

Similarly, most studies report no differences in ophidiomycosis as a function of sex. For example, there was no sex-related difference in prevalence in water snakes in two separate studies (*Nerodia* sp.) [15,53]. Further, a retrospective study of captive snakes from the Smithsonian National Zoological Park indicated the presence of ophidiomycosis in snake specimens, and there were no sex differences in prevalence [56]. We also found few sex differences; there were no differences in (1) prevalence of ophidiomycosis for all tested snakes, or in percentage of *O. ophidiicola* positivity of swabs of the head, body or sores, (2) the percentage of snakes with lesions, (3) the percentage of lesions that were positive, and (4) the mean number of lesions (refer to Table 1). These are the measures usually examined in field studies. However, we did find a significant difference in ophidiomycosis status from year to year, and in the number of years snakes encountered following a positive test. Females more often switched from positive to negative, and they lived longer following testing positive.

The status switches (from positive to negative, or negative to positive) are perhaps one of the more interesting findings of this study because, although researchers have reported that snake populations show a decline in prevalence of ophidiomycosis from the time snakes leave hibernation to the fall in one season, few studies examined individuals from one year to the next, or for an extended number of years. Haynes et al. [57] did report a few snakes that switched from year to year. The studies that mention switches seasonally do not report either the age or sex of the snakes and have low sample sizes. We suggest that this is a fruitful area of research that might show sex differences with regard to exposure, susceptibility, or response (e.g., clearance).

### 4.3. Ophidiomyces ophidiicola Endemism in Northern Pine Snakes in New Jersey

Although many papers identify ophidiomycosis as a “Disease of Emerging Concern”, more recent papers suggest otherwise because of its wide distribution in the wild [18,21,22,23]. It has been identified in nearly every state east of Mississippi [15,17] and in Canada [21]. Moreover, Lorch et al. [58] confirmed cases of *O. ophidiicola* positivity in museum specimens as early as 1945 in the U.S. However, museum specimens from Texas showed that although clinical signs of infection were constant over time, the presence of *O. ophidiicola* increased in time and space [15]. The Texas data clearly indicated that *O. ophidiicola* was an emerging, novel pathogen. We suggest that it is likely that whether one considers ophidiomycosis to be endemic (or not) may be a matter of when in time analyses for *O. ophidiicola* were conducted and whether museum specimens were available for analysis. For example, Haynes et al. [57] examined ophidiomycosis prevalence in several locations (Lake Erie watersnakes, *Nerodia sipedon insularum*), showing spatial and temporal variations. They suggested that their data indicated *O. ophidiicola* endemism at most sites, but emergence in one. Such large-scale studies can indicate spatial and temporal patterns.

Our pine snake study has been on-going for 50 years, and the hibernation excavation studies for 40 years [35,38,39], providing longitudinal data on individuals ranging in age from hatchlings to 25 years in any one year, albeit with small sample sizes. Over that time, we regularly recorded if snakes had severe lesions; our notes indicate that in the 1980s, 1990s, and early 2000s there were few obvious lesions (only 0–3 snakes/year had a lesion). They were recorded as “hibernation sores” (Burger, Unpublished data [35]). The presence of lesions became more obvious in the mid-2010s—perhaps indicating that there was a low level of infection (and thus endemism) all along. Alternatively, the low level of lesions reflected that they may be simply lesions unrelated to *O. ophidiicola* infection or any other infection.

What our study adds to the discussion is that not only is ophidiomycosis endemic in the Northern pine snake population as a whole in snakes examined every year (2018–2023) in hibernation [25], but that surface skin swabs of individuals indicated that they switch back and forth from year to year. What is unclear is whether snakes are forever infected once they test positive, and the disease is dormant internally, or whether they are reinfected each new time they test positive. Another key factor was that individual pine snakes had fewer lesions with each succeeding year, suggesting that individual snakes are combating disease. Examining the number of lesions may be a useful epidemiological measure for ophidiomycosis, and we are investigating this further. Since many pine snakes hibernate together in underground tunnels and chambers, and the soil below harbors viable *O. ophidiicola* [37], it may be that the snakes get reinfected while in hibernation, but over time are more resistant and develop fewer sores. Whether ophidiomycosis is emergent or endemic in this population may not be the most important issue, the key issues are whether there are adverse health effects, associated reductions in survival, and population declines.

### 4.4. Survival Post-Ophidiomycosis

Another major contribution of our study is the realization that once infected, Northern pine snakes can live for at least eight years post-infection, and it is the only study to monitor individuals for this many years. Studies in laboratory settings have shown that snakes inoculated with *O. ophidiicola* show the same clinical signs as snakes that were found positive for *O. ophidiicola* in the wild [11,14]. For example, McKenzie et al. [14] performed a detailed experiment using corn snakes that showed that inoculation with *O. ophidiicola* resulted in similar symptoms of lesions as in wild snakes. Temperature and brumation affected prevalence of *O. ophidiicola* (qPCR tested) as well, and 87% of their inoculated snakes died. Such experiments are extremely helpful in demonstrating both pathogenesis and the potential for contributing to population losses.

Many studies examine whether wild snakes test positive for ophidiomycosis in different seasons of one year, or even in the next year, but individuals are not routinely studied. Clark et al. [20] reported severe population effects on an isolated population of rattlesnakes. Similarly, Tetzlaff et al. [27] monitored 17 Massasauga rattlesnakes (*Sistrurus catenatus*) over three years (few were repeated), and reported that one individual survived ophidiomycosis for three winters, but survival or changes in *O. ophidiicola* status were not objectives of that study. Moreover, they reported that no snakes that were positive and tested several times ever tested negative, which is contrary to our findings. However, Lind et al. [28] followed marked pygmy rattlesnakes (not known-aged) for two years, and found that snakes switched from positive to negative, within and among years, but they did not record survival with ophidiomycosis other than from one year to the next. They did, however, find that infected males (as determined by rating of clinical signs) had lower testosterone levels compared to unaffected males during the summer spermatogenesis and breeding seasons. Further, the continuation of that study to 5 years indicated that there were significant increases in thermoregulatory behavior and ecdysis that could reduce the time and energy devoted to foraging and reproductive behavior, although responses of individuals were not reported [59]. McKenzie et al. [60] also examined short-term survival of free-ranging watersnakes and queensnakes (*Regina septemvittata*) and did not find any mortality effects of ophidiomycosis over a one-year period in 16 individuals (although they did find increased surface basking and movement). These behavioral effects are certainly important components of reproductive success and survival that require further study for more than a few years, in marked individuals, in more locations and in more species.

For most snake species, survival in the wild is estimated, and the ages of wild-caught individuals are not known. Obtaining data on *O. ophidiicola* positivity in known-aged individuals is very difficult. For example, there is little information about survival in pine snakes other than our studies. However, Gacheny et al. [61] reported on one male adult pine snake (SV 1342 mm) that was monitored for 12 years, and one juvenile female pine snake (SV 653 mm) that was monitored for 10 years (Georgia). Our long-term study, using data from snakes captured in hibernacula, indicated that survival was not significantly different for males and females, although females had higher philopatry to hibernacula— that is, females were often present for many years in a row—but the overall survival from hatching to last presence in the hibernacula was not different for males and females: females simply had more sequential data ([35], Burger Unpublished Data).

The oldest pine snake that we tested for *O. ophidiicola* was positive at 25 years of age, and the next oldest was 20 years old. Some of the snakes in the present study that were still alive 8 years after they first tested positive may still be alive (Figure 6). Further, some of the snakes tested in 2018 were still alive in 2025. For example, the first three examples in Table 1 illustrate individuals that were still alive in the last year or two of the study; at that time they were 15, 16, and 18 years old (some of the oldest snakes ever found ([35], Burger, Unpublished Data). Data from our long-term dataset indicate that only 12% of pine snakes ever reach 8 years of age. Being able to examine known-aged snakes after reaching breeding age (e.g., adults, 3–5 years) was only possible because of our long-term mark/recapture pine snake hibernation studies [35,50].

The sign that most snakes in our study had ophidiomycosis was the presence of skin lesions of varying severity coupled with qPCR results. In the 40 years of our hibernation study, we have never found a pine snake in a hibernaculum that had severe ophidiomycosis, had noticeable unhealthy body condition, or died from a fungal infection. Snakes that died within our hibernacula were mainly hatchlings that had been squashed by heavier snakes lying on top of them in a chamber; a 1200 gr snake on top of a 50 gr hatchling can be fatal [35]. Without finding any physical manifestations of an “illness” (other than lesions), and with no sick, dying or dead pine snakes in our hibernacula, it is hard to imagine that ophidiomycosis is responsible for the population decline that we have reported elsewhere [35]. As Clark et al. [20] noted, declines in populations, such as some isolated timber rattlesnakes, are likely a function of several factors, including disease, loss of genetic diversity, and climate change. Still, ophidiomycosis may pose a risk to some snake species globally [62]. Further, when we capture female pine snakes when they are laying eggs or at other times in the summer or fall, there are almost no clinical signs of ophidiomycosis (no lesions, bumps on the head), and those tested were not positive for *O. ophidiicola* [25]. The effects of other environmental variables and climate change need to be further examined and may have effects on individuals and the overall use of hibernacula. The effects of ophidiomycosis on survival require additional study, over a wider geographical range, but the presence of *O. ophidiicola* did not indicate a significant effect on the individuals reported in this study and likely did not affect the viability of the population examined.

## 5. Conclusions

Our examination of the prevalence, severity, and survival post-infection with *O. ophidiicola* indicates that understanding the complexities of disease requires long-term studies with individuals of different species, in different geographical locations, for multiple years. Northern pine snakes in our study had high philopatry to specific hibernacula, had high prevalence rates of *O. ophidiicola* positivity, often alternated whether they were positive or not, showed few sex or age relationships with positivity or number of lesions, and could live at least 8 years after first being infected. The average number of lesions did not seem to relate to age or sex. The apparent resistance to severe manifestations of ophidiomycosis (e.g., *O. ophidiicola* positivity), and the demonstration of pine snakes being positive one year and negative the next, provides data relevant to understanding the effects of the disease on long-term population viability of this species. That females had a higher rate of testing negative after being positive than males requires further study, because females have higher fidelity to hibernations sites and might be expected to have higher infection rates, given that hibernacula are locations of transmission among snakes. Females bearing a lower cost of ophidiomycosis might bode well for the long-term effects of ophidiomycosis on pine snake populations. The presence of the same rates of testing positive for *O. ophidiicola,* and the fact that snakes test positive for *O. ophidiicola* over many years (up to 8 years after testing positive for *O. ophidiicola*) in the same individuals, suggest that ophidiomycosis is endemic in the population. The apparent lack of age-related differences in survival after testing positive for *O. ophidiicola*, along with the lack of an increased number of positive sores with age, suggests that *O. ophidiicola* is not severely affecting these pine snakes.

After the end of the study, we found one snake still alive in 2026. One female (315) was still alive 9 years post-infection.

## Figures and Tables

**Table 1:** Individual pine snakes’ patterns of ophidiomycosis in the New Jersey Pinelands. M = missing in the hibernaculum that year, and X means the snake was present but qPCR testing for *O. ophidiicola* was not done in 2024 and 2025. H = head; V = ventral; C = cloaca; Any = a snake with a positive test for any sample. N (green) = tested negative for *O. ophidiicola, *P (red) = tested positive for *O. ophidiicola. *Colors were provided to create visual contrast of qPCR test results. Each block represents one snake encountered during up to eight years from 2018 (first year of sampling) to 2025. Snake number refers to the last three digits of snakes’ PIT tag numbers. Ages of snakes were known because snakes were monitored prior to our ophidiomycosis studies.

#	Year	Sex	Age	Any	H	V	C	# Lesions		Notes
								Pos	Neg	
1	2018	F	8	P	P	P				Initially, snake 315 was positive in 2019, followed by 2 years with all negative samples. In 2022 tests were all positive except for cloaca, and all tests were negative in 2023. It lived at least 2 more years and had one lesion in 2024. This illustrates the importance of continuing to follow individuals to determine the long-term effect of ophidiomycosis.
	2019	F	9	P	N		N	1	1	
	2020	F	10	N	N	N	N	0	1	
	2021	F	11	N	N	N	N	0	1	
	2022	F	12	P	P	P	N	2	0	
	2023	F	13	N	N	N	N			
	2024	F	14		X	X	X			
	2025	F	15		X	X	X			
2	2018	M	12	N	N	N	N			Despite being at the same hibernation site (in the same years) as the female above, male snake 281 was negative for the first 2 years. It was missing in 2020 but was positive in 2021. It was found in only 4 of 7 years, and positive during only 2 of those years.
	2019	M	13	N	N	N	N			
	2020	M	14	M	M	M	M			
	2021	M	15	P	N	P	P	3	0	
	2022	M	16	M	M	M	M			
	2023	M	17	P	N	N		2	3	
	2024	M	18		X	X	X			
3	2020	F	11	P	P	P	P	1	0	For this snake (female 620), in 2020 all swabs were positive, but in 2021 and 2023 only one lesion was positive. She lived the next two years and was found in hibernation but not tested (although she had 2 lesions in 2024).
	2021	F	12	P	N	N	N	1	0	
	2022	F	13	M	M	M	M			
	2023	F	14	P	N	N		1	0	
	2024	F	15		X	X	X			
	2025	F	16		X	X	X			
4	2019	F	0	N	N	N				Female 817 was negative in 2019, and missing from hibernation the next year. She was negative in 2021, while most swabs were positive in 2022 and 2023, with many lesions present.
	2020	F	1	M	M	M	M			
	2021	F	2	N	N	N	N			
	2022	F	3	P	P	P	P	5	1	
	2023	F	4	P	N	P		6	0	
5	2018	F	11	N	N	N	N			Female 123 tested negative (except for 1 positive lesion in 2019) for 2 years. She was positive in 2022 (with many lesions) and had five lesions in 2023. She was alive in 2024 but died in late winter, likely from a forest fire. She was followed for 7 years.
	2019	F	12	P	N	N	P			
	2020	F	13	N	N	N	N	0	1	
	2021	F	14	N	N	N	N			
	2022	F	15	P	P	P	P	5	0	
	2023	F	16	P	P	P	P			
6	2018	M	12	N	N	N				Male 281 was negative for 2 years, missed a year of sampling (2020) and then tested positive in 2021 and 2023. This male was missing for 2 years and present in the 7th year (2024) but not tested.
	2019	M	13	N	N	N	N			
	2020	M	14	M	M	M				
	2021	M	15	P	N	P	P	3	0	
	2022	M	16	M	M	M				
	2023	M	17	P	N	N		2	3	
7	2018	M	8	N	N	N				Male 359 illustrates a different pattern. He was negative at the beginning of the study (2018) and turned up negative 5 years later.
	2019	M	9	M	M	M				
	2020	M	10	M	M	M				
	2021	M	11	M	M	M				
	2022	M	12	M	M	M				
	2023	M	13	N	N	N		0	1	
8	2019	M	14	N	N	N	N	0	2	For male 836, all samples were negative the first year; then this male had only 1 positive lesion the next year, but more positive samples in the following two years (4 years of data).
	2020	M	13	P	N	N	P	0	1	
	2021	M	16	P	N	N	N	4	0	
	2022	M	17	P	N	P	N	2	0	

## Data Availability

The original contributions presented in the study are included in the article, further inquiries can be directed to the corresponding author.

## References

[1] Skerratt L.F., Berger L., Speare R., Cashins S., McDonald K.R., Phillott A.D., Hines H.B., Kenyon N. (2007). Spread of chytridiomycosis has caused the rapid global decline and extinction of frogs. EcoHealth.

[2] Allender M.C., Dreslik M., Wylie S., Phillips C., Wylie D.B., Maddox C., Delaney M.A., Kinsel M.J. (2011). *Chrysosporium* sp. infection in Eastern Massasauga rattlesnakes. Emerg. Infect. Dis..

[3] Martel A., Spitzen-van der Sluijs A., Blooi M., Bert W., Ducatelle R., Fisher M.C., Woeltjes A., Bosman W., Chiers K., Bossuyt F. (2013). *Batrachochytrium salamandrivorans* sp. Nov. causes lethal chytridiomycosis in amphibians. Proc. Nat. Acad. Sci. USA.

[4] Hoyt J.R., Langwig K.E., White J.P., Kaarakka H.M., Redell J.A., Kurta A., DePue J.E., Scullon W.H., Parise K.L., Foster J.T. (2018). Cryptic connections illuminate pathogen transmission within community networks. Nature.

[5] Blehert D.S., Hicks A.C., Behr M., Meteyer C.U., Berlowski-Zier B.M., Buckles E.L., Coleman J.T.H., Darling S.R., Gargas A., Niver R. (2009). Bat white-nose syndrome: An emerging fungal pathogen?. Science.

[6] Frick W.F., Pollock J.F., Hicks A.C., Langwig K.E., Reynolds D.S., Turner G.G., Butchkoski C.M., Kunz T.H. (2010). An emerging disease causes regional population collapse of a common North American bat species. Science.

[7] Spitzen-van Der Sluijs A., Spikmans F., Bosman W., De Zeeuw M., Van Der Merij T., Goverse E., Kik M., Pasmans F., Martel A. (2015). Rapid enigmatic decline drives the fire salamander (*Salamandra salamandra*) to the edge of extinction in the Netherlands. Amphibia-Reptilia.

[8] Gibbons J.W., Scott D.E., Ryan R.J., Buhhmann K.A., Tuberville T.D., Metts B.S., Greene J.L., Mills T., Leiden Y., Poppy S. (2000). The global decline of reptiles: Déjà vu amphibians. BioSci.

[9] Allender M.C., Raudabaugh D.B., Gleason F.H., Miller A.N. (2015). The natural history, ecology, and epidemiology of *Ophidiomyces ophidiicola* and its potential impact on free-ranging snake populations. Fungal Ecol..

[10] Allender M.C., Hileman E.T., Moore J., Tetziaff S. (2016). Detection of *Ophidiomyces ophidiicola*, the causative agent of Snake Fungal Disease, in the Eastern Massassauga (*Sistrurus catenatus*) in Michigan. J. Wildl. Dis..

[11] Lorch J.M., Lankton J., Werner K., Falendysz E.A., McCurley K., Blehert D.S. (2015). Experimental infection of snakes with *Ophidiomyces ophidiicola* causes pathological changes that typify snake fungal disease. mBio.

[12] Lorch J.M., Knowles S., Lankton J.S., Mitchell K., Edwards J.L., Kapfer J.S., Staffen R.A., Wild E.R., Schmidt K.Z., Ballmann E.A. (2016). Snake fungal disease: An emerging threat to wild snakes. Phil. Trans. R. Soc. B.

[13] Allender M.C., Baker S., Wylie D., Loper D., Dreslik M.J., Phillips C.A., Maddoz C., Driskell E.A. (2015). Development of snake fungal disease after experimental challenge with *Ophidiomyces ophidiicola* in Cottonmouths (*Agkistrodon piscivorous*). PLoS ONE.

[14] McKenzie C.M., Oesterle P.T., Stevens B., Shirose L., Mastromonaco G.F., Lillie B.N., Davy C.M., Jardine C.M., Nemeth N.M. (2020). Ophidiomycosis in Red Cornsnakes (*Pantherophis guttatus*): Potential roles of brumation and temperature on pathogenesis and transmission. Vetern. Pathol..

[15] Harding S., Moretta-Urdiales M., Nordmeyer M.S., Worstl E., Rodriguez D.E. (2022). Leveraging preserved specimens of *Nerodia* to infer the spatiotemporal dynamics of *Ophidiomyces ophidiicola* via Quantitative Polymerase Chain Reaction. Nat. Ecol. Evol..

[16] Dillion R.M., Paterson J.E., Manorome P., Ritchie K., Shirose L., Slavik E., Davy C.M. (2022). Seasonal and interspecific variation in the prevalence of *Ophidiomyces ophidiicola* in Ophidiomycosis in a community of free-ranging snakes. J. Wildl. Dis..

[17] Harding S.F., Becker G., Yates J.R., Crump P., Forstner M.R.J., Mullin J.M., Rodriguez D. (2022). Comparative host-pathogen associations of snake fungal disease in sympatric species of water snakes (*Nerodia*). Sci. Rep..

[18] Baker S.J., Haynes E., Gramhofer M., Standord K., Bailey S., Christman M., Conley K., Frasca S., Ossiboff R.J., Lobato D. (2019). Case definition and diagnostic testing for snake fungal disease. Herpetol. Rev..

[19] McKenzie J.M., Price S.J., Fleckenstein J.L., Drayer A.N., Connette G.M., Bohuski E., Lorch J.M. (2019). Field diagnostics and seasonality of *Ophidiomyces ophidiicola* in wild snake populations. EcoHealth.

[20] Clark R.W., Marchand M.N., Clifford B.J., Stechert R., Stephens S. (2011). Decline of an isolated timber rattlesnake (*Crotalus horridus*) population: Interactions between climate change, disease, and loss of genetic diversity. Biol. Conserv..

[21] Davy C.M., Shirose L., Campbell D., Dillon R., McKenzie C., Nemeth N., Braithwaite T., Cai H., Degazio T., Dobbie T. (2021). Revisiting Ophidiomycosis (Snake Fungal Disease) after a decade of targeted research. Front. Vet. Sci..

[22] Haynes E., Chandler H.C., Stegenga B.J., Adamovicz L., Ospinal E., Zerpa-catanhorn D., Stevenson D.J., Allender M.C. (2020). Ophidiomycosis in surveillance of snakes in Georgia, USA reveals new host species and taxonomic associations with disease. Sci. Rep..

[23] Burger J., Jeitner C., Zappalorti R.T., Bunnell J., Ng K., DeVito E., Schneider D., Gochfeld M. (2025). Snake fungal disease (Ophidiomycosis) in Northern Pine Snakes (*Pituophis melanoleucus melanleucus*) in New Jersey: Variations by year, sex, and morphological sampling site. J. Fungi.

[24] Allender M.C., Baker S., Britton M., Kent A.D. (2018). Snake fungal disease alters skin bacterial and fungal diversity in an endangered rattlesnake. Sci. Rep..

[25] Burger J., Gochfeld M., Zappalort R., Bunnell J., Jeitner C., Schneider D., Ng K., DeVito E., Lorch J.M. (2023). Prevalence of *Ophidiomyces ophidiicola* and epizootiology of snake fungal disease in free-ranging Northern Pine Snakes (*Pituophis melanoleucus melanoleucus*) in New Jersey. Environ. Monit. Assess..

[26] Allain S.J.R., Duffus A.L.J., Marschang R.E. (2022). Editorial: Emerging infections and diseases of herpetofauna. Front. Vet. Med..

[27] Tetzlaff S.J., Ravesi M.J., Allender M.C., Carter E.T., DeGregorio B.A., Josimovich J.M., Kingsbury B.A. (2017). Snake fungal disease affects behavior of free-ranging Massasauga rattlesnakes (*Sistrurus catenatus*). Herpetol. Conserv. Biol..

[28] Lind C.M., McCoy C.M., Farrell T.M. (2018). Tracking outcomes of snake fungal disease in free-ranging pygmy rattlesnakes (*Sistrurus miliarius*). J. Wildl. Dis..

[29] Mark M., Christensen T.C., Kwait R.E., Eskew E.A., Zoccolo I., Struck E.J., Maslo B. (2024). Apparent Ophidiomycosis alters Eastern copperhead (*Agkistrodon contortrix*) behavior and habitat use. J. Wildl. Dis..

[30] Duffus A.L.J., Hughes D.F., Kautz A., Allain S.J.R., Meshaka W.E. (2022). Repeated sampling of wild individuals reveals *Ophidiomyces ophidiicola* infection dynamics in a Pennsylvania snake assemblage. J. Wildl. Dis..

[31] Fuchs L.D., Tupper T.A., Aguilar R., Lorentz E.B., Bozarth A., Fernandez D.J., Lawlor D.M. (2020). Detection of *Ophidiomyces ophidiicola* at two mid-Atlantic natural areas in Anne Arundel County, Maryland and Fairfax County, Virginia, USA. Amphib. Reptl. Conserv..

[32] Lictira D., Quinn D.P., Reeder J.E., Gavitt G., Dickson J., Hess B., Mangold J.J., Tuttle A.D., Roses-Rosas A., Frasca S. (2019). Snake fungal disease in Colubridae snakes in Connecticut, USA in 2015 and 2017. J. Wildl. Dis..

[33] Burger J., Jeitner C., Zappalort R., Bunnell J., Ng K., DeVito E., Schneider D., Schneider D., Gochfeld M. (2023). Snake fungal disease in free-ranging Northern Pine Snakes (*Pituophis melanoleucus melanoleucus*) in New Jersey: Lesions, severity of sores, and investigator’s perceptions. J. Fungi.

[34] Lind C.M., Lorch J.M., Moore I.T., Vernasco B.J., Farrell T.M. (2018). Seasonal sex steroids indicate reproductive costs associated with snake fungal disease. J. Zool..

[35] Burger J., Zappalorti R.T., Gochfeld M. (2025). Natural History of the Northern Pine Snake: Fifty Years of Research on Its Ecology, Behavior, and Conservation in New Jersey’s Pinelands.

[36] Golden D.M., Winkler P., Woener P., Fowles G., Pitts W., Jenkins D. (2009). Status Assessment of the Northern Pine Snake (Pituophis m. melanoleucus) in New Jersey: An Evaluation of Trends and Threats.

[37] Campbell L.J., Burger J., Zappalorti R.T., Bunnell J.F., Winseler M.E., Taylor D.R., Lorch J.M. (2021). Soil reservoir dynamics of *Ophidiomyces ophidiicola*, the causative agent of snake fungal disease. J. Fungi.

[38] Burger J., Zappalorti R.T., Gochfeld M., Boarman W., Caffrey M., Doig V., Garber S., Mikowsky M., Safina C. (1988). Hibernacula and summer dens of Pine Snakes (*Pituophis m. melanoleucus*) in the New Jersey Pine Barrens. J. Herpetol..

[39] Burger J., Zappalorti R.T. (2011). The Northern Pine Snake (Pituophis melanoleucus) in New Jersey: Its Life History, Behavior and Conservation.

[40] Burger J. (2019). Vulnerability of Northern Pine Snakes (*Pituophis melanoleucus,* Daudin, 1809) during fall den ingress in New Jersey, USA. Amphib. Rept. Conser..

[41] Burger J., Zappalorti R.T. (2015). Hibernation site philopatry in Northern Pine Snake (*Pituophis melanoleucus*) in New Jersey. J. Herpetol..

[42] Burger J., Zappalorti R.T. (2016). Conservation and protections of threatened Northern Pine Snake (*Pituophis melanoleucus*) in New Jersey Pine Barrens, USA. Herpetol. Conserv. Biol..

[43] McBride M.P., Wojick K.B., Georoff T.A., Kimbro J., Garner M.M., Wang X., Childress A.L., Wellehan F.X. (2015). *Ophidiomyces ophidiicola* dermatitis in eight free-ranging timber rattlesnakes (*Crotalis horridus*) from Massachusetts. J. Zool. Wildl. Med..

[44] Bohusky E., Lorch J.M., Griffin K.M., Blehert D.S. (2015). TaqMan real-time polymerase chain reaction for detection of *Ophidiomyces ophidiicola*, the fungus associated with snake fungal disease. Vet. Res..

[45] Siegel S.E. (1956). Nonparametric Statistics.

[46] Statistical Analysis Systems (SAS) (2020). Statistical Analysis.

[47] McDonald J.H. (2022). Fisher’s Exact Test of Independence. Handbook of Biological Statistics—On Line. https://www.biostathandbook.com/fishers.html#:~:text=Fisher%27s%20exact%20test%20is%20more,test%20for%20larger%20sample%20sizes.

[48] Sperry J.H., Wolff P.J., Melder C.A., Nevarez J.G., Huskins S.D., Pearce S.E. (2023). Habitat use, activity pattens, and survival of Louisiana pinesnakes (*Pituophis ruthveni*) in West-central Louisiana. Southeast. Nat..

[49] United States Fish and Wildlife Service (USFWS) (2016). Threatened species status for the Louisiana pinesnake. Fed. Regist..

[50] Burger J., Zappalorti R.T., Gochfeld M. (2018). Hatchling survival to breeding age in Northern Pine Snake (*Pituophis melanoleucus)* in the New Jersey Pine Barrens: Human effects on recruitment from 1986 to 2017. PLoS ONE.

[51] Hill A.J., Leys J.E., Bryan D., Erdman F.M., Malone K.S., Russell G.M., Applegare R.D., Fenton H., Niedringhaus K., Miller A.N. (2018). Common cutaneous bacteria isolated from snakes inhibit growth of *Ophidiomyces ophidiicola*. EcoHealth.

[52] Pare J.A., Sigler L. (2016). An overview of reptile fungal pathogens in the genera *Nannizziopsis Paranannizziopsis*, and *Ophidiomyces*. J. Herpetol. Med. Surg..

[53] Haskins D.L., Brown M.K., Melchner K., Colemand A.L., Allender M.C., Tuberille T.D. (2024). Factors predicting apparent Ophidiomycosis in wild Brown Watersnakes (*Nerodia taxispiota*). J. Wildl. Dis..

[54] Long R.B., Love D., Seeley K.E., Patel S., Allender M.C., Garner M.M., Ramer J. (2019). Host factors and testing modality agreement associated with Ophidiomyces infection in a free-ranging snake population in southeast Ohio, USA. J. Zoo. Wildl. Med..

[55] Guthrie A.L., Knowles S., Ballmann A.E., Lorch J.M. (2016). Detection of snake fungal disease due to *Ophidiomyces ophidiicola* in Virginia, USA. J. Wildl. Dis..

[56] Anderson K.B., Steeil J.C., Neiffer D.L., Evans M., Peters A., Allender M.C., Cartoceti A.N. (2021). Retrospective review of Ophidiomycosis (*Ophidiomyces ophidiicola*) at the Smithsonian’s National Zoological Park (1983–2017). J. Zoo. Wildl. Med..

[57] Haynes E., Stanford K., Gremhofer M., Vivirito K., Duranta K., Wright A., Varga C., Allender M.C. (2022). Epidmiology of Ophidiomycosis in Lake Erie watersnakes (*Nerodia sipedon insularum*). J. Wildl. Dis..

[58] Lorch J.M., Price S.J., Lankton J.S., Drayer A.N. (2021). Confirmed cases of *Ophidiomyces* in museum specimens from as early as 1945, United States. Emerg. Infect. Dis..

[59] Lind C.M., Agugiaro J., Lorch J.M., Farrell T.M. (2023). Ophidiomycosis is related to seasonal patterns of reproduction, ecdysis, and thermoregulatory behavior in a free-living species. J. Zool..

[60] McKenzie J.M., Price S.J., Connette G.M., Bonner S.J., Lorch J.M. (2021). Effects of snake fungal disease on short-term-survival, behavior, and movement of free-ranging snakes. Ecol. Applic..

[61] Gacheny M., Howze J.M., Smith L.L. (2022). Survival Records of Free-ranging Southeastern USA Snakes. Herpetol. Rev..

[62] Di Nicola M., Coppari L., Notomista T., Marini D. (2022). *Ophidiomyces ophidiicola* Detection and infection: A global review on a potential threat to the world’s snake populations. Eur. J. Wildl. Res..

